# Fluoride Exposure Induces Inhibition of Sodium-and Potassium-Activated Adenosine Triphosphatase (Na^+^, K^+^-ATPase) Enzyme Activity: Molecular Mechanisms and Implications for Public Health

**DOI:** 10.3390/ijerph16081427

**Published:** 2019-04-21

**Authors:** Declan Timothy Waugh

**Affiliations:** EnviroManagement Services, 11 Riverview, Doherty’s Rd, P72 YF10 Bandon, Co. Cork, Ireland; declan@enviro.ie; Tel.: +353-23-884-1933

**Keywords:** Na^+^, K^+^-ATPase, fluoride; molecular mechanisms of inhibition, Na^+^, K^+^-ATPase and pathological states, cognitive impairment, neurological diseases, metabolic diseases, lung diseases, cancer

## Abstract

In this study, several lines of evidence are provided to show that ${\mathrm{Na}}^{+}$, $K^{+}$-ATPase activity exerts vital roles in normal brain development and function and that loss of enzyme activity is implicated in neurodevelopmental, neuropsychiatric and neurodegenerative disorders, as well as increased risk of cancer, metabolic, pulmonary and cardiovascular disease. Evidence is presented to show that fluoride (F) inhibits ${\mathrm{Na}}^{+}$, $K^{+}$-ATPase activity by altering biological pathways through modifying the expression of genes and the activity of glycolytic enzymes, metalloenzymes, hormones, proteins, neuropeptides and cytokines, as well as biological interface interactions that rely on the bioavailability of chemical elements magnesium and manganese to modulate ATP and ${\mathrm{Na}}^{+}$, $K^{+}$-ATPase enzyme activity. Taken together, the findings of this study provide unprecedented insights into the molecular mechanisms and biological pathways by which F inhibits ${\mathrm{Na}}^{+}$, $K^{+}$-ATPase activity and contributes to the etiology and pathophysiology of diseases associated with impairment of this essential enzyme. Moreover, the findings of this study further suggest that there are windows of susceptibility over the life course where chronic F exposure in pregnancy and early infancy may impair ${\mathrm{Na}}^{+}$, $K^{+}$-ATPase activity with both short- and long-term implications for disease and inequalities in health. These findings would warrant considerable attention and potential intervention, not to mention additional research on the potential effects of F intake in contributing to chronic disease.

## 1. Introduction

Sodium, potassium-activated adenosine triphosphatase (${\mathrm{Na}}^{+}$, $K^{+}$-ATPase) is an integral protein in the plasma membrane that transports Na^+^-ions to the outside and K^+^-ions to the inside of the cell at the expense of ATP, and thus maintains sodium and potassium homeostasis in animal cells [1,2]. ${\mathrm{Na}}^{+}$, $K^{+}$-ATPase (NKA) is responsible for the electrochemical gradient across the plasma membrane, a prerequisite for electrical excitability and secondary transport in neurons, as well as for the transport of other ions and metabolites necessary for the regulation of the cellular ionic homeostasis [3]. In addition, to its function in maintaining cell homeostasis, NKA activity plays a crucial role in the function of neurotransmitter transporters essential for regulating neurotransmitter signaling and homeostasis [4]. By using the energy from ATP to establish asymmetric distributions of ions across the cell membrane, NKA couples metabolic energy to cellular functions and to signaling events both between and within cells [5].

**Figure 1:**
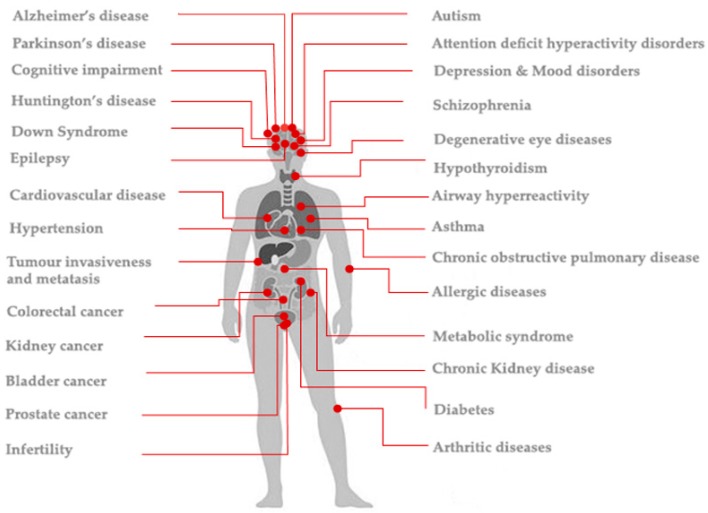
Pathophysiological conditions and neurological disorders associated with loss of Na^+^, K^+^-ATPase activity.

Given the importance of NKA in cellular homeostasis and intracellular signaling, impairment or downregulation of NKA activity has been implicated in many pathophysiological conditions, including asthma and allergic diseases, metabolic disorders, cancer, cardiovascular and degenerative brain diseases, as well as neuropsychological disorders [3,6,7,8,9,10,11,12,13,14], as illustrated in Figure 1 and discussed below. 

Previous studies have shown that inhibition of NKA activity has been found to accelerate depletion of adenosine triphosphate (ATP), induce mitochondrial depolarization, suppress reactive oxygen species (ROS) scavenging, and enhance ROS production and oxidative stress [15,16,17]. It is known that a causal relationship has been identified between NKA enzyme inhibition and airway hyperreactivity [18]. Consistent with this, NKA inhibition is associated with asthma [19,20] and chronic obstructive pulmonary disease (COPD) [21]. Furthermore, loss of NKA activity is associated with allergic diseases [22,23] including allergic rhinitis [24] and blood diseases including thalassemia and sickle cell anaemia [25]. Extensive studies show that NKA is essential for sperm mobility and male fertility [26,27,28,29]. In addition, loss of NKA activity is also associated with rheumatoid arthritis [30,31], metabolic syndrome [32], including; chronic kidney disease [33,34,35]; diabetes mellitus [36,37]; diabetic nephropathy and cardiomyopathy [38,39,40]; cardiovascular complications [32,41,42,43]; hypertension [40,44,45,46,47,48,49,50,51,52] and obesity [11]. Loss of NKA activity is also implicated in degenerative eye diseases including cataract formation and age related macular degeneration [53]. In addition to inflammatory disorders as noted previously, loss of NKA activity has been found to be associated with tumour invasiveness, metastasis, and tissue fibrosis [54], kidney cancer [54], prostate cancer [55], bladder cancer [56] and urothelial cancer [57]. Consistent with these findings showing an association between loss of NKA in carcinoma and cancer progression, an isoform of the β subunit of NKA has been found to be a tumour-suppressor [58] and its expression along with total NKA activity has been found to be markedly reduced in prostate cancer [55] and kidney cancer [59]. Moreover, the adhesion molecule on glia (AMOG), another isoform of β subunit of NKA has been found to inhibit glioma cell invasion, while its downregulation increases invasion in glial cells [60]. Downregulation of the alpha 1 subunit of NKA has also been implicated in colorectal cancer [61].

Furthermore, a great body of evidence associates neurotoxicity with a reduction of NKA activity, suggesting that reduction in NKA activity may be a link between several common neurotoxic mechanisms [14,15,62,63,64,65,66,67,68,69,70,71,72,73,74,75,76,77,78,79]. Loss of NKA is associated with autism [65,66,69]; Alzheimer’s disease [66,80,81]; Parkinson’s disease [82]; amyotrophic lateral sclerosis [70,82]; Down syndrome and Huntington’s disease [69]; depression and mood disorders [71,72,73,74]; bipolar disorder [14,75,76] and schizophrenia [15,77,79], as well as in animal models of depression [83,84]. Interestingly, NKA activity has been found to be significantly lower in subjects with phenylketonuria [85], a disease associated with intellectual disability, seizures, behavioural problems and mental disorders. Of note, loss of NKA activity has also been found to be associated with neonatal seizures and epilepsy [86,87]. It is also known that recovery of NKA activity in the hippocampus is responsible for neuroprotection [88]. Experimental studies have also shown that impairment of NKA activity in neonatal brain of rats leads to increased anxiety-like behaviour and memory impairment. Interestingly, in this study treatment with folic acid was found to reverse the inhibition of NKA and alleviated cognitive deficits associated with enzyme inhibition [89]. As NKA activity is essential for synaptic and neural functionality [3] and since NKA has been shown to trigger dendritic growth [90]; hence, it is possible that reduction in the activity of this enzyme may disrupt normal brain development. In this context, it has been demonstrated in-vivo that NKA inhibition causes selective neuronal loss in rodents [91]. In addition, it has been demonstrated that the extent of neuronal loss observed roughly paralleled inhibition of the enzyme [92]. It has been reported loss of NKA activity causes impairments in the sodium pump leading to neuronal hyperexcitability in the CNS [10,93,94]. NKA inhibition also increases neuronal susceptibility to glutamate excitotoxicity contributing to neurotoxicity [95,96]. Furthermore, NKA impairment has been found to downregulate the synaptic α-amino-3-hydroxy-5-methyl-4-isoxazolepropionic acid (AMPA) receptor, leading to synaptic transmission defects and cognitive impairment [97]. Of note, researchers have also identified glutamatergic neurotransmission dysregulation and cognitive dysfunctions in major psychiatric disorders: including, schizophrenia, depressive disorders and suicidal behaviours [98,99,100,101]. Excess extra-cellular glutamate leading to excitotoxicity has also been suggested to play a role in in numerous neurodegenerative diseases including amyotrophic lateral sclerosis, Alzheimer’s disease and Huntington’s disease [102].

A relationship between glutamate excitotoxicity and neuroinflammation in autism has also been ascertained [103,104]. Moreover, in animal studies, loss of AMPA receptors have been found to result in early-onset motor deficits, hyperactivity, cognitive defects, behavioural seizures and sleep disorders [105]. Taken together, these findings suggest a possible causal link between loss of NKA activity and childhood neurodevelopmental disorders that present with motor deficits, hyperactivity and cognitive defects including attention deficit hyperactivity disorder (ADHD) and autism spectrum disorders (ASD). Moreover, it is important to note that ADHD is highly comorbid with other psychiatric or neurodevelopment disorders associated with loss of NKA including, major depressive disorder and schizophrenia [106,107], which again supports the hypothesis that loss of NKA is implicated in the pathophysiology of ADHD. Importantly, NKA is also critical for sodium iodide symporter (NIS) functionality and iodine transport [108,109,110]. Thus, NKA inhibition may contribute to iodine deficiency [110]. Iodine deficiency can lead to hypothyroidism [111,112]. Interestingly, hypothyroidism has also been found to decrease NKA activity [113,114]. Crucially, Schmitt et al. found that hypothyroidism at a critical period of development in utero can result in permanent inhibition of NKA activity [114]. Moreover, Ahmed et al. showed that the hypothyroid status during pregnancy and lactation produced inhibitory effects on NKA as well as Ca(2+)-ATPase and Mg(2+)-ATPase in different brain regions of the offspring [115]. Further studies have established that hypothyroidism leads to marked reduction in dendritic branching in the rat brain [116], which is consistent with loss of NKA activity as previously described.

It is well established that NKA activity is inhibited by fluoride [117,118,119,120,121,122,123,124,125,126,127,128,129,130,131,132,133,134,135,136,137,138]. Furthermore, the results from human studies provide a biological gradient by which serum fluoride levels inhibit NKA activity in adults [137,138]. Considering the exclusion criteria and number of participants in these latter studies, these findings are significant. Notably, the inhibitory effect was found to occur in adults at serum fluoride levels < 5.0 µM and the inhibitory effects increased significantly as serum F levels increased in a dose dependent manner. [137,138]. For example, Arulkumar et al. observed that at serum ionic fluoride levels of 14.75 µM the activity of NKA declined by approximately 60 per cent compared to controls [137]. Considering that NKA activity is inhibited by hypothyroidism, the potential role of water fluoridation or fluoride intake with respect to increased prevalence of hypothyroidism must not be overlooked [139,140]. Interestingly, Kheradpisheh et al. in a case control study recently found that fluoride levels in drinking water has impacts on thyroid hormones even in the standard concentration of less than 0.5 mg/L. Among healthy participants without thyroid disease median TSH levels were found to increase when drinking water fluoride levels increased from 0–0.29 mg/L to 0.3–0.5 mg/L. Moreover, among subjects with clinically diagnosed hypothyroidism the effects of fluoride intake from drinking water on TSH were highly significant [140]. This finding is consistent with the Peckham study in England, which reported an increased prevalence of hypothyroidism, in communities where drinking water was artificially fluoridated [139]. As previously described, loss of NKA activity has been implicated in the pathogenesis of neurodevelopmental, neuropsychiatric and neurodegenerative disorders, as well as increased risk of cancer, metabolic, pulmonary and cardiovascular disease. Therefore, it is plausible that fluoride inhibition of NKA may have previously unforeseen consequences on public health and health inequalities, particularly in countries where drinking water is artificially fluoridated. However, studies examining the molecular mechanisms by which fluoride may contribute to such health inequalities are lacking. This relationship is particularly important given that exposure to fluoride can occur through water, food and other common sources such as dental products, in addition to occupational and environmental exposures. Although much information has become available in recent decades, the molecular mechanisms of fluoride inhibition of NKA remain to be defined. The lack of detailed information on the molecular mechanisms underlying how fluoride inhibits NKA activity impedes our understanding of how fluoride exposure may also contribute to pathological states. Consequently, the objective of this current study is to elucidate the molecular mechanisms and biological pathways by which fluoride inhibits NKA activity. In addition, this study examines the potential implications of fluoride induced loss of NKA activity on human health and disease inequalities.

## 2. The Role of Fluoride in Oral Health and Sources of Fluoride Exposure

While the focus of this study is directed toward the molecular mechanisms underlying how fluoride inhibits NKA, it is also necessary to discuss a number of important findings from the literature regarding the role of fluoride (F^−^) in oral health and sources of F^−^ exposure, in order to better understand the importance of cumulative exposures.

It is acknowledged that F^−^ has no known essential function in human growth and development and no signs of F^−^ deficiency have been identified [141]. However, F^−^ is considered to have played a major role in the reduction of dental caries in the past decades in the industrialized countries. It is added as an anti-caries agent to a variety of vehicles, particularly drinking water and toothpastes. Though F^−^ is not essential nutrient, it has been recognized for some time that topical, rather than systemic exposure of F^−^ controls carious lesion development [142,143,144,145]. However, caries development is not a F^−^ deficiency disease [141]. Interestingly, a key mechanism in the anti-caries effect of F^−^ is the direct inhibition of ATPases activity in oral bacteria [146,147,148,149].

Today, community water fluoridation and F^−^ toothpaste are considered the most common sources of F^−^ exposure in the USA [142,150]. In countries such as Ireland, the UK, Australia and New Zealand, where habitual tea drinking is commonplace, the major dietary source of F^−^ is tea consumption [151,152,153]. In addition to tea, fluoridated water, and toothpaste other sources of F^−^ exposure include other beverages produced from fluoridated water (powdered infant formula, fruit juices, soft drinks, coffee, beers); pesticide residues in foods, foods processed or cooked in fluoridated water; foods grown in soil containing F^−^ or irrigated with fluoridated water; consumption of foods with elevated F^−^ levels (i.e., seafood and processed chicken); foods cooked in Teflon cookware; tobacco consumption; use of fluoridated mouthwash; use of medical inhalers containing fluoridated gases, and fluoridated medications, in addition to other environmental or occupational exposures to F^−^ [153]. Although the amount of F^−^ ingested in diet can be theoretically measured in dietary intake studies, by measuring the F^−^ content in foods and beverages, the most reliable and accurate method of measuring F^−^ exposure is by measuring the F^−^ content in serum/plasma, bone or urinary F^−^ levels. As NKA activity is present in blood and considering the evidence that F^−^ levels in blood inhibit NKA activity, it is therefore necessary to learn more about how different sources of F^−^ exposure contribute to blood F^−^ levels in humans. Moreover, understanding the routes of F^−^ exposure in infancy is essential when considering the long-term health implications of chronic F^−^ exposure on NKA activity and implications for health and well-being long term. Furthermore, the magnitude of exposure can be examined by comparing breast fed infants in low F^−^ communities to formula fed infants in fluoridated communities, as shown in Table 1 below.

The F^−^ level in breast milk from mothers in a low F^−^ community where drinking water F^−^ levels less than 0.16 mg/L are 0.004 mg/L and 0.009 mg/L in breast milk from mothers residing in communities where drinking water F^−^ levels are 1.0 mg/L [154]. Elsewhere it has been reported that the concentration of F^−^ in cows’ milk is ten-fold higher than human milk and typically ranges from 0.03 to 0.06 ppm [155]. However, past studies have shown that F^−^ concentrations in cow’s milk can vary significantly depending on the F^−^ level of water provided to dairy herds. For example, Gupta et al. demonstrated that when the F^−^ concentration in drinking water was 0.47, 0.82 and 1.32 mg/L the F^−^ concentrations in cow’s milk was 0.016, 0.074 and 0.18 mg/L respectively [156]. Interestingly, a recent study conducted by researchers at Newcastle University and Teesside University in England reported that the mean F^−^ content in whole milk products available in a fluoridated region of the UK was 0.08 ppm [157]. This data indicates that dairy herds in the region were provided with mains fluoridated water as a source of drinking water. Moreover, previous studies have reported that F^−^ values in cow’s milk ranging from 0.1–0.4 mg/L are consistent those found in F^−^ poisoned dairy herds [158]. From the above data it is obvious that the source of F– in drinking water provided to dairy herds can influence the F^−^ content in milk used for the manufacture of powdered infant formula. It has also been reported that the F^−^ content in powdered infant formula products can vary depending on the source of water used in processing and that the use of optimally fluoridated mains can result in higher F^−^ levels in powdered infant formula products. Thus, in certain western countries where drinking water is fluoridated significant variations in the F^−^ content in powdered infant formula products have been reported [159,160,161]. However, it is widely acknowledged the main contributor to the F^−^ intake in infants is the F^−^ content in water used to reconstitute powdered infant formulas. It should also be noted that formulas mixed with optimally fluoridated water provide the highest mean F^−^ daily intake [162].

In areas naturally low in water F^−^ and dietary F^−^ intake exclusively breast-fed infants aged less than 12 months have been reported to have a mean ionic serum F– levels of 0.22 μM [163]. By contrast, in fluoridated communities in the United States the mean F^−^ level in infants aged 4–6 months and 7–12 months has been reported to be 4.22 ± 3.7 µM, and 1.56 ± 0.53 µM, respectively [164]. Moreover, the mean ionic plasma F^−^ level in infants with renal failure during their first 18 months of life was 6.3 µM compared to 3.16 µM in age matched controls [164]. As is evident from this study, plasma F^−^ levels varied significantly among infants aged 4–6 months with the maximum F^−^ levels being approximately 8 µM. The variations reported reflect the peak plasma F^−^ levels associated with proximity to feeding and the use of breast milk versus optimally fluoridated water used to reconstitute powdered infant formulas. It must be emphasized that the F^−^ levels reported in this study are not unexpected, as a previous study conducted by Anderson et al. in the Republic of Ireland reported that the consumption of infant formula reconstituted with fluoridated water may result in F^−^ doses above the recommended tolerable upper intake level for healthy adults [165]. A current study conducted in the USA further supports this observation. In this study, it was reported that the use of optimally fluoridated water (0.7 mg/L) in the preparation of infant formula resulted in 36.8% of infants exceeding the UL [166]. Furthermore, in this study, it was reported that among bottle fed infants the highest bioavailability of F^−^ occurs in the first six months of life [166]. Importantly, this also coincides with the period when F^−^ excretion is impaired in infants due to immature kidney function [167,168]. Thus, the decline in plasma F^−^ concentrations after 6 months of age observed by Warady and associates coincides with the development of renal function and increased urinary excretion of F^−^. Further studies conducted in another fluoridated city in the USA reported that when infants were fed milk-based formula reconstituted with non-fluoridated water, the mean plasma F^−^ concentrations two hrs post feeding was 0.77 μM, despite the lack of direct exposure to fluoridated water [169]. This finding reflects the higher plasma F^−^ levels in mothers in fluoridated than non-fluoridated communities. Furthermore, this study demonstrated that a dose of 0.25 mg of F^−^ administered to twenty infants aged one to eighteen months two hours after their last feed resulted in mean peak plasma F^−^ levels of 3.3 µM (range 2.52–4.85 µM). Consistent with the findings of Warady and associates, the authors of this latter study acknowledged that the F^−^ intake and exposure for infants fed powdered infant formula reconstituted with fluoridated water would be significantly higher than those reported in their study, though for some unexplained reason they did not attempt to measure ionic plasma F^−^ levels in infants provided powdered infant formula reconstituted with tap water in a community with fluoridated drinking water [169].

**Table 1:** Fluoride levels in human milk, cow’s milk and infant formula and serum/plasma fluoride levels in infants less than 12 months of age in fluoridated and non-fluoridated communities.

	Non Fluoridated Region mg/L	Fluoridated Region mg/L	Reference
**Human milk**	$\bar{x}=$ 0.004	$\bar{x}=$ 0.009	[154,161]
**Cow’s Milk**	$\bar{x}=$ 0.016	0.074–0.18	[156]
**Cow’s milk based powdered**	0.02–0.18	0.49–1.40	[159]
**infant formula reconstituted**			
**with tap water**			
	**Non-Fluoridated**	**Fluoridated**	**Reference**
	**Ionic F levels**	**Ionic F levels**	
	**µM**	**µM**	
**Fully Breast-fed infants**			
1–6 months	$\bar{x}$ = 0.22		[163]
**Formula fed**			
1–6 months	$\bar{x}$ = 0.29		[163]
**Breast fed with semi solids**			
6–12 months	$\bar{x}$ = 0.35		[163]
	(0.10–0.67)		
**Aged 1 month**		0.89	[169]
**Aged 7 months**		0.53	[169]
**Breast and formula fed**			
Aged 4–6 months		$\bar{x}=$ 4.33	[164]
		(0.52–8.0)	
Aged 7–12 months		$\bar{x}=$ 1.56	[164]
with semi solids		(1.03–2.1)	
Aged 4–18 months		$\bar{x}=\;3.16$	

A mention should be made that the F^−^ levels in infants residing in a fluoridated community in the USA as measured by Warady and associates are within the range observed to cause inhibition of NKA activity in adults [137,138]. Indeed, the levels are also higher than what has been observed among workers occupationally exposed to F^−^ in aluminum smelting factories at the end of the working day in Sweden [170] and Japan [171]. Notably, Ehrnebo and Ekstrand reported that the mean plasma F^−^ levels in workers at the end of their shift were 2.54 μM [170], while Kono reported serum F^−^ levels ranging from 2.21 to 3.47 µM among exposed workers [171]. Moreover, the ionic F^−^ levels in blood in infants reported by Warady and associates are within the range known to be associated with skeletal and non-skeletal fluorosis in humans. A review of literature addressing ionic F^−^ levels associated with skeletal and non-skeletal fluorosis in humans has been the subject of an earlier study by Waugh et al. [153]. However, it should be also noted that the F^−^ levels reported by Warady and associates for infants aged 4–6 months were two fold higher than those reported for 60-day old non-diabetic weaning Wistar rats fed drinking water with a F^−^ concentration of 50 mg/L, while the plasma F^−^ levels in infants with chronic kidney disease were comparably to diabetic rats fed drinking water with 50 mg/L [172]. Toxicity studies using 3-week old weaning male Wistar rats provided F^−^ in drinking water at concentrations of 50 mg/L for 60 days lead to mean ionic plasma F^−^ levels of 3.78 µM [173], which is similar to reported for (A/J) mice given 50 mg/L F^−^ in drinking water for 11 weeks (mean serum F^−^ levels of 3.31 µM) [174]. Collectively, these findings suggest that in fluoridated community’s plasma F^−^ levels in maternal cord blood and human neonates in early infancy are comparable to the plasma F^−^ levels in rodents administered drinking water with a F^−^ level of approximately 50 mg/L.

Moreover, studies involving adult human subjects in countries without water fluoridation have demonstrated that the consumption of single cup of tea containing between 1.4 and 2 mg/L F^−^ result in peak plasma F^−^ levels of approximately 3–4 µM. [175,176]. It is necessary to note that the frequency of F^−^ dosage is known to affect plasma F^−^ levels due to the terminal plasma half-life of F^−^. Thus, multiple low dose exposures can result in higher steady state plasma F^−^ levels than single dose exposure to higher doses [177,178,179]. Hence, habitual consumption of tea can result in skeletal fluorosis [153]. Moreover, currently about one-fifth of the currently marketed pharmaceuticals are organofluorine compounds and almost one-third of the top 100 top-selling drugs are organofluorine compounds. Fluoridated pharmaceuticals include antidepressants, anti-inflammatory agents, antimalarial drugs, antipsychotics, antiviral agents and steroids [180]. While there is a paucity of information on the biotransformation of fluoridated pharmaceuticals in general, several synthetic organic fluoride drugs which have been found to undergo high rates of biotransformation and defluorination resulting in significantly elevated plasma F^−^ levels and in some instances chronic F^−^ intoxication in humans. While it is beyond the scope of this present study to review the literature on fluoridated pharmaceuticals, of the fluoridated drugs currently on the market, Voriconazole is acknowledged to cause chronic F^−^ intoxication, resulting in musculoskeletal chronic pain disorders and skeletal fluorosis [181,182]. Skiles et al. reported that elevated plasma F^−^ levels of 24.3 μM after 6 months of voriconazole treatment resulted in skeletal fluorosis. Once F^−^ toxicity was confirmed and voriconazole was discontinued, within 3 weeks plasma F^−^ level declined to 6.7 μM [181]. Furthermore, the use of fluoridated anaesthesia such as sevoflurane can provide 20 times the total daily dietary intake from all sources of fluoridated food and water combined [183], resulting in peak plasma F^−^ levels in the range of 50 μM [184].

Dental-care products are also a major source of F^−^ exposure especially for children, because many tend to use more toothpaste than is advised, their swallowing control is not as well developed as that of adults, and many children under the care of a dentist undergo fluoride treatments [150]. The use of topical fluoride gels which contain high concentrations of F^−^ have been reported to significantly increase children’s F^−^ plasma levels up to 79 μM after treatment. Among adults the use of F^−^ gels can result in peak plasma F^−^ levels of 51 μM [185,186]. Researchers found that the peak concentration was normally reached within 1 to 2 h of treatment and remained significantly elevated for up to 14 h [185]. The most commonly used fluoride-containing dental product is toothpaste. The vast majority of toothpastes sold today contains between 1000 ppm and 1500 ppm. Human studies have demonstrated that ingested F^−^ in toothpaste is readily absorbed into systemic circulation resulting in rapid rise in plasma ionic F^−^ levels [187]. A recent study involving healthy adults aged between 20–35y residing in a non-fluoridated community in England observed that when subjects were given a non-fluoridated toothpaste plasma F^−^ levels, as measured in the morning after toothbrushing declined significantly from approximately 3.2 µM to 0.67 µM, respectively [188]. In another study conducted among adults residing in non-fluoridated community in Scotland, it was found that brushing twice a day (morning and evening) with toothpaste containing 1000 ppm and 1500 ppm for four weeks was found to increase plasma F^−^ levels at midday to 1.18 and 1.33 µM respectively, compared to 0.7 µM in subjects using non-fluoride toothpaste [189]. The differences in plasma F^−^ levels observed in these latter studies can be accounted for the half-life of F^−^ in systemic circulation. After a single dose of F^−^ plasma concentrations rise to a peak within one hour and decrease back to baseline with a half-life of two-three hours [176]. Therefore, taking the time differences in sampling of blood the results observed by Zohoori et al. and Jacobson are almost identical. Taken together, this data shows that a single brushing with F^−^ toothpaste in the morning can provide a dose of F^−^ comparable to consuming a cup of black tea with a F^−^ content of 1.4–2.0 mg/L [175,176]. Similar to tea, case reports also indicate that skeletal fluorosis can occur from excessive use of F^−^ toothpaste [190]. Kurtlan et al. reported a case study of an adult American male aged 52 yrs who developed skeletal fluorosis from brushing his teeth six times per day. Laboratory evaluation of blood found serum F^−^ levels ranged from 15 to 18.0 µM [190]. The subject did not reside in a fluoridated community and had no known occupational or environmental exposure to F^−^ apart from toothpaste. The patient stated that he did not swallow toothpaste, used non-fluoridated mouthwash, had semi-annual dental visits, but without F^−^ treatments, did not drink tea or wine, and had not chewed tobacco, inhaled snuff, or cooked with Teflon pots. Within 8 months of documentation of skeletal fluorosis and after avoiding fluoridated dental products, serum F^−^ decreased to < 2.5 µM [190]. It is also important to note that the European Academy of Paediatric Dentistry (EAPD) recommend that children who are below the age of two should use toothpastes with low fluoride concentrations (less than 500 ppm) [191]. For children aged 2–7 years, a pea sized amount of F^−^ dentifrice (0.25 g) has been recommended [192,193,194]. However, several studies examining toothpaste usage by children aged 3–6 years have consistently shown that the amount of toothpaste used on toothbrushes typically varies from 0.35 to 3.5 g which can lead to excessive F^−^ intake [194,195], particularly among children who brush twice daily or more. A current study in a fluoridated community in England involving young children aged 4 to 6-years of age residing reported that the contribution of toothpaste to total dietary F^−^ intake was 53% [196]. In this study, the highest total daily F^−^ intake (4.439 mg/day = 0.22 mg/kgbw/day) was for a 5-year-old child of which 72% (3.217 mg/day = 0.16 mg/kgbw/day) was from toothpaste ingestion [196]. In summary, the above studies illustrate that in evaluating the effects of F^−^, consideration must be given to effects of cumulative exposures and their contribution to total F^−^ intake and ionic plasma F^−^ levels. As NKA is found in plasma and bound to plasma membranes, it is the ionic F^−^ levels in systemic circulation and within cells that interacts with NKA functionality. The remainder of this study addresses NKA regulation and the molecular mechanisms by which F^−^ inhibits enzyme activity and the most significant health risks likely to be associated with F^−^-induced inhibition of enzyme activity.

## 3. Na^+^, K^+^-ATPase Regulation by Phosphorylation/Dephosphorylation

NKA is a plasma membrane embedded protein in all animal cells. NKA consists of two noncovalently linked α and β subunits. The alpha subunit is also known as the catalytic or functional subunit, since it contains the binding sites for protein kinase, protein phosphatase and transmembrane ion transport activities [197,198,199]. The catalytic α-subunit is responsible for conversion of ATP energy to transport of Na^+^ and K^+^ across cell membranes and has ATP and cardiac glycosides binding sites. The β-subunit is responsible for delivery and insertion of alpha one in cell membranes [200,201,202]. Thus, plasma membrane expression of the NKA requires the assembly of its alpha- and β- subunits [3] and the β subunit must interact with α subunit in order to accomplish ion transport [203]. The presence of magnesium facilitates the binding of ATP to NKA thereby providing the chemical energy required for ion channel function and secondary active transport [2]. Functionally, it has been identified that the activity of NKA is inhibited by phosphorylation. Furthermore, cyclic adenosine monophosphate (cAMP)-dependent protein kinase, (PKA) and protein kinase C (PKC) catalyse the phosphorylation of the enzyme [204]. It is well proven that PKC phosphorylates the NKA α subunit, leading to a decrease in enzyme activity [205,206,207,208,209,210,211,212,213].

## 4. Molecular Mechanisms by which Fluoride Inhibits Na^+^, K^+^-ATPase Activity

**Figure 2:**
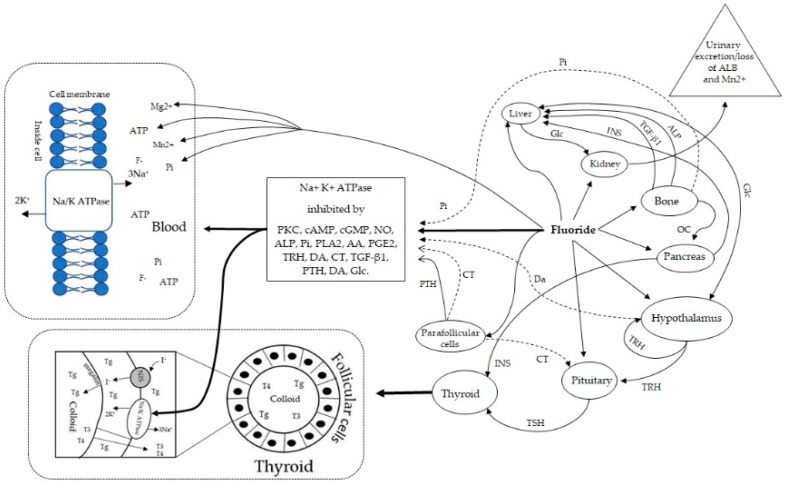
Schematic representation of the molecular mechanisms and biological pathways by which fluoride inhibits Na^+^, K^+^-ATPase activity on plasma membranes and in thyroid follicular cells.

Despite the large number of studies demonstrating that F^−^ inhibits NKA activity, the molecular mechanisms of down-regulation are not yet clearly understood [214]. To address the limitations in understanding of the molecular mechanisms of down-regulation it was first necessary to identify modulatory mechanisms from published literature. Thus, a review of literature was undertaken using PubMed and other search engines (Google, Google Scholar, ResearchGate, Yahoo) to source pertinent research articles and publications. Second, in order to elucidate the molecular mechanisms by which F^−^ may inhibit NKA activity, evidence was sought from published literature including human, animal and in vitro studies to examine how F^−^ interacted with each of the biological pathways identified. As illustrated in Figure 2 and summarized in Table 2, NKA activity is downregulated by a variety of hormones, proteins, metalloenzymes, neuropeptides and cytokines. Furthermore, regulatory mechanisms governing circulating inorganic phosphate, glucose homeostasis and adenosine-triphosphate production play a crucial role in downregulating enzyme activity.

### 4.1. The Role of Protein Kinase RNA-like ER Kinase (PERK) in Regulating Na^+^, K^+^-ATPase Activity and the Influence of Fluoride on PERK Activity

As previously described, the β subunit of NKA regulates both the activity and the conformational stability of α subunit and plasma membrane expression of the NKA requires the assembly of its α-and β-subunits. Thus, inhibition of the β subunit leads to reduced expression and lower enzyme activity. It is also important to note that NKA β subunit expression and maturation requires the interaction of Wolfram Syndrome 1 (WFS1) protein [215]. Furthermore, it has been shown that reduced expression of WFS1 results in reduced expression of NKA β subunit [202]. It has been reported that WFS1 expression is induced by protein kinase RNA-like ER kinase (PERK) [216]. Recent in vivo studies examining the role of genetics in response to F^−^ exposure found that chronic F^−^ exposure inhibits the expression of PERK [217]. Taken together, this data suggests that F^−^ may also inhibit WFS1 protein expression, leading to inhibition of NKA expression and activity.

### 4.2. The role of Protein Kinase C (PKC) in Regulating Na^+^, K^+^-ATPase Activity and the Influence of Fluoride in Regulating PKC Activity

Phosphorylation is a widely used, reversible means of regulating enzymatic activity [218]. It is further known that phosphorylation of the NKA catalytic subunit inhibits enzyme activity [204]. Protein kinase C (PKC) is critical to phosphorylation [219] and as previously described it is well proven that PKC phosphorylation of the α subunit of NKA leads to a decrease in its enzyme activity [206,207,208,209,210,211,212,213]. Conversely, inhibition of PKC has been found to stimulate NKA enzyme activity [220,221].

Interestingly, Bocanera et al. also reported that PKC pathway inhibits thyroid iodide uptake in calf thyroid cells by an action distal to cAMP generation and probably because of a decrease in NKA activity [222]. As previously described, hypothyroidism has also been found to decrease the activity of NKA [113,114,115]. and there is evidence from human studies that water fluoridation and elevated F^−^ levels in drinking water may contribute to hypothyroidism [139,140]. Hypothyroidism has also been shown to significantly upregulate PKC expression and activity [223,224]. Furthermore, it is also well established that activation of PKC is a physiological action of F^−^-induced cellular toxicity [133,225,226,227,228,229,230]. Overall, these data suggest that F^−^ activation of PKC is a key mechanism underlying F^−^ inhibition of NKA activity and that the contributory effect of F^−^ exposure to hypothyroidism may further potentiate inhibition.

### 4.3. The Role of Cyclic Adenosine-Monophosphate (cAMP) in Regulating Na^+^, K^+^-ATPase Activity and the Influence of Fluoride in Regulating cAMP

The possibility that cAMP or cyclic AMP may play a role in regulation of NKA activity was reported three decades ago [231]. Subsequent studies have confirmed that cAMP inhibits NKA activity [232,233,234]. Although not completely elucidated, it is important to examine the molecular mechanisms by which this inhibition occurs. The precise mechanism of action will be defined in in the following section elucidating the molecular mechanism by which F^−^ alters cAMP and adenosine-triphosphate (ATP) bioavailability.

It is also established that F^−^ increases the conversion of ATP to cAMP by stimulation of adenylyl cyclase [235,236]. Consistent with this, evidence from human and animal studies show that F^−^ stimulates cAMP production [237,238,239,240,241,242,243,244,245,246,247,248] leading to increased concentrations of cAMP in plasma, saliva, urine, and tissues. Moreover, in vitro human tissue models have demonstrated that F^−^ in micromolar concentrations of 1–10 µM significantly increases the synthesis of cAMP in a dose dependent manner [249]. In addition, thyroid-stimulating-hormone (TSH) is known to be a potent stimulant of adenylyl cyclase activity resulting in increased synthesis of cAMP [250,251]. Furthermore, early in vitro studies by Wolf and Jones documented the largely identical effect of F^−^ and TSH on adenyl cyclase activity resulting in increased stimulation of cAMP [252]. Consistent with this, several in vitro studies have shown a synergistic effect of F^−^ and TSH on adenylate cyclase activity resulting in increased cAMP production [253,254]. Evidence from human studies also show that F^−^ induces TSH production [140,255,256,257,258,259,260,261,262,263,264,265,266,267,268,269]. In this scenario, a positive feedback loop exists that may amplify the effects of TSH and F^−^ on cAMP activity, which may explain the synergistic effect of F^−^ and TSH on cAMP production.

In elucidating the molecular mechanisms by which increased production of cAMP inhibits NKA activity, it is necessary to emphasise that the conversion of ATP to cAMP leads to a reduction in ATP molecules. Since energy supply is a limiting factor controlling NKA activity, ATP depletion leads to inhibition of enzyme activity. As described above, F^−^ increases the conversion of ATP to cAMP. Further studies have shown that F^−^ inhibits ATP bioavailability [270,271]. Indeed, studies have shown that F^−^ induces depletion of ATP in erythrocytes (red blood cells) [271]. Consistent with these findings, several studies have reported that ATP depletion induced by F^−^ leads to inhibition of NKA in erythrocytes [126,128]. Furthermore, it is known that the breakdown of glucose by glycolysis is a major source of ATP [272]. Enolase is required to allow glycolysis to proceed [273] and inhibition of enolase leads to depletion of ATP [274]. Given that it is well established that F^−^ inhibits glycolysis via inhibition of enolase [275,276,277,278]. it is not surprising that recent in vitro studies found that inhibition of glycolysis by F^−^ contributed to reduced NKA activity [136]. However, it is important to also note that F^−^ inhibition of enolase is significantly stronger in the presence of phosphate [278]; the significant of which will be discussed later in this study. Taken together, this data demonstrates that F^−^ stimulation adenylyl cyclase and TSH secretion leads to increased synthesis of cAMP reducing the bioavailability of ATP thereby contributing to inhibition of enzyme activity. Furthermore, it seems reasonable to suggest that the mechanisms outlined above elucidate why previous studies found that hypothyroidism was associated with lower NKA activity [113,114,115].

However, in addition to the role of cAMP in depleting ATP, it has also been reported that cAMP inhibits NKA activity by enhancing phosphorylation of the NKA α subunit [279]. As previously described, it is well proven that phosphorylation of the α subunit, leads to a decrease in its enzyme activity [206,207,208,209,210,211,212,213]. Therefore, strong evidence indicates that the action of F^−^ in upregulating cAMP may lead to inhibition of NKA activity by two distinct pathways, by reducing ATP bioavailability and increasing phosphorylation of the α subunit of NKA.

### 4.4. The Influence of Magnesium in Regulating Na^+^, K^+^-ATPase Activity and the Influence of Fluoride on Magnesium Homeostasis

The human body contains around 25 g of magnesium [280]. Magnesium is necessary for the functioning of over 300 enzymes in humans [281], with 90% of total body magnesium being contained in bones and muscles (~63% and ~27%  respectively), 90% of which is bound and with only 10% being free [282]. In the serum, 32% of magnesium is bound to albumin, whereas 55% is free [280]. As previously discussed, Jorgensen et al. elucidated that the presence of magnesium facilitates the binding of ATP to NKA, thereby providing the chemical energy required for ion channel function and secondary active transport [2].

F^−^ is known to form complexes with magnesium and increased concentrations of magnesium reduce F^−^ absorption in the gastrointestinal tract [150]. This action would also result in reduced absorption of magnesium. Experimental studies have shown that low magnesium diets significantly enhance F^−^ absorption [283] and F^−^ accumulation in calcified tissue retards the mobilization of skeletal magnesium [284]. Furthermore, as magnesium is bound to albumin, loss of albumin in urine can lead to enhanced excretion of magnesium and lower levels of magnesium in systemic circulation. In line with this, I recently elucidated how the loss of albumin in urine and resultant hypoalbuminemia (low levels of albumin in blood) contribute to iodine deficiency and that F^−^ acts to induce increased urinary excretion of albumin and hypoalbuminemia [110]. Therefore, evidence would strongly suggest that F^−^ exposure can lead to magnesium deficiency. Consistent with these hypothesis, human studies have shown that F^−^ significantly reduced serum magnesium along with other trace metals including manganese, copper and zinc [285,286,287]. In agreement, animal studies have also found that F^−^ exposure results in decreased serum magnesium [288,289,290,291,292]. Thus, the above data suggests that F^−^ can inhibit NKA activity by lowering serum magnesium thereby impairing ATP binding to NKA.

### 4.5. The Influence of Calcineurin, Calmodulin and Manganese in Regulating Na^+^, K^+^-ATPase Activity and the Influence of Fluoride on Calcineurin, Calmodulin and Manganese Homeostasis

It has also been reported that the dephosphorylation (activation) of NKA is mediated by calmodulin-dependent calcineurin (Cn), a serine/threonine phosphatase [204,208,293]. Therefore, inhibition of Cn will lead to downregulation of NKA activity. Although the molecular pathway has not been completely elucidated, it has also been reported that Cn is inhibited by F^−^ [294,295,296]. However, it is known that Cn binds to calmodulin (CaM) and the complex of Cn and CaM is the active species of the phosphatase. Furthermore, aside from CaM, Cn requires an additional divalent metal ion such as manganese for structural stability and full activity [297]. Manganese is also a crucial activator of Cn activity [298,299].

It has been shown that F^−^ upregulates the activation of adenylate cyclase by CaM [300] and activation of adenylate cyclase increases the intracellular level of cAMP [301]. As previously described, adenylate cyclase is the enzyme which catalyses the conversion of ATP to cAMP, thus increased cAMP synthesis reducing the bioavailability of ATP. As previously noted, cAMP has been observed to inhibit NKA activity and the molecular mechanisms of inhibition have been described. However, CaM has also been found to inhibit NKA activity. in human red blood cells [302]. Furthermore, previous studies have shown that CaM can mediate the phosphorylation of acetylcholine receptor and CaM stimulation of phosphorylation was enhanced most in the presence of F^−^ [303]. Revealingly, studies have shown that the presence of F^−^ inhibited the activation of phosphodiesterase by CaM [304]. Interestingly, phosphodiesterase is responsible for the hydrolysis (breakdown) of cAMP [305], a crucial finding that may elucidate why F^−^ exposure is associated with increased concentrations of cAMP.

Furthermore, chronic F^−^ intake has been shown to significantly reduce serum manganese levels in humans along with other trace metals including magnesium, copper and zinc [286]. Lower manganese accumulation in skeletal tissue has also been observed in humans suffering from fluorosis [306]. In agreement, several animal studies have also found that excessive F^−^ exposure resulted in decreased manganese in serum [292,307,308], as well as in reduced manganese in liver, kidney, heart, lung and muscle tissue [309,310,311,312]. Collectively, this data suggests that F^−^ enhances the activation of adenylate cyclase by CaM leading in increased production of cAMP. Furthermore, the ability of F^−^ to enhance the phosphorylation activity of CaM suggests that F^−^ may contribute to the inhibitory effects of CaM on NKA enzyme activity. In addition, the contributory effect of F^−^ reducing manganese bioavailability suggests that this action may also be a potential mechanism in F^−^ inhibition of Cn activity. As previously described, manganese is a crucial activator of Cn and also binds to Cn ensuring structural stability and full enzyme activity. Thus, lower manganese reduces Cn expression. Furthermore, as the dephosphorylation of NKA is mediated by Cn, reduced expression of Cn or inhibition of Cn activity contributes to enhanced phosphorylation, which in turn leads to inhibition of enzyme activity. 

### 4.6. The Role of Cyclic Guanosine Monophosphate (cGMP) and Nitric Oxide in Regulating Na^+^, K^+^-ATPase Activity and the Contribution of Fluoride to Regulating cGMP and Nitric Oxide

It is also known that an important intracellular second messenger cyclic guanosine monophosphate (cGMP) inhibits NKA activity. [231,313,314,315]. The seminal study by de Oliveira et al. demonstrated cGMP inhibited NKA activity and that cGMP activation was induced by nitric oxide (NO) [315]. Accordingly, NO generating compounds play an important role in regulating NKA activity. Consistent with this finding, several studies have observed that NO inhibits the molecular activity of renal NKA [232,313,316,317,318,319]. Several studies have also shown that F^−^ induces NO synthesis in vivo [320,321,322,323,324,325,326]. Furthermore, experimental studies have shown that F^−^ stimulates cGMP in the kidney and thyroid [327,328]. Hence, the above data suggests that F^−^ inhibits NKA activity by stimulating NO expression and cGMP activity.

### 4.7. Cytokine TGF-β1 Inhibits NKA Activity

Transforming growth factor β 1 or TGF-β1 has been found to inhibit the expression of the β-subunit of NKA required for enzyme function [55]. As previously noted, plasma membrane expression of the NKA requires the assembly of its α- and β- subunits [3] and the β subunit must interact with α subunit in order to accomplish ion transport [203]. Hence, TGF-β1 inhibition of β-subunit expression leads to inhibition of NKA expression and protein activity.

It has been reported that TGF-β1 plays an important role in fluorosis and increased levels of TGF-β1 have been suggested as an important marker in the evaluation of the pathological action of F^−^ in bone tissue [329,330]. In vivo and in vitro experimental studies of fluorosis have shown that F^−^ upregulates TGF-β1 protein and mRNA expression in bone cells [329,331,332,333]. Importantly, calcitonin (CT), a hormone that is secreted by parafollicular cells of the thyroid gland, has been found to be a potent stimulator of TGF-β1 protein synthesis as well as TGF-β1 mRNA expression [334]. Furthermore, a large body of evidence from epidemiological studies has demonstrated that F^−^ is a potent inducer of CT expression in humans [335,336,337,338,339,340]. Of fundamental importance, is the seminal research by Chen and associates in providing a biological dose-exposure response relationship for F^−^ exposure in humans on CT expression at relatively low F^−^ intakes. Notably, in this study, it was demonstrated that differential expression of CT occurs when urinary F^−^ levels exceeded 0.38 mg/L [338]. Taken together, this data suggests that the contribution of F^−^ in enhancing the expression of TGF-β1 via upregulation of CT is another mechanistic pathway whereby F^−^ inhibits NKA mRNA expression and protein activity.

### 4.8. The Role of Inorganic Phosphate in Regulating Na^+^, K^+^-ATPase Activity

In light of the fact that phosphorylation inhibits NKA activity, rationality postulates that elevated levels of inorganic phosphate (Pi) will also act as an inhibitor of NKA activity. In support of this hypothesis, it has previously been reported that Pi plays a role in phosphorylation of NKA [341] and high concentrations of Pi inhibit enzyme activity [342]. Furthermore, previous studies have also shown that Pi inhibits phosphatase activity of calcineurin (Cn) [343]. As previously described, dephosphorylation of NKA is mediated by Cn [2]. Hence, evidence suggests that higher levels of Pi may contribute to inhibition of NKA activity by decreasing dephosphorylation activity of Cn. Moreover, it is well established that alkaline phosphatase (ALP) is an enzyme responsible for catalysing dephosphorylation of phosphate esters, which leads to liberating Pi [344]. As ALP levels rise; more Pi is liberated [345]. Thus, ALP enzymatic activity plays a role in regulating Pi levels. This also suggests that ALP must play a role in regulating NKA functionality. ALP is found in normal osteoblasts, and the mode of action of F^−^ in stimulating APL activity in humans is well recognized [242,346,347,348,349,350,351]. It is also well established that F^−^ acts to increase osteoblast formation [150]. It has been demonstrated in vitro that F^−^ at concentrations as low as 0.1 μM increase the expression of ALP and stimulate the proliferation of osteoblasts [352]. Furthermore, it is known that ALP activity increases in the presence of CT [353].

As previously described, F^−^ is a potent inducer of CT. Moreover, it is also known that the protein RANKL (receptor activator of nuclear factor-KB ligand) increases osteoclast number, bone resorption, and subsequently Pi release [354,355]. Furthermore, Pi uptake by osteoclasts require sodium-dependent phosphate (Na-Pi) transporters that are dependent on ATPase activity. The energy requirement to drive this process is high, thus, a large amount ATP is required [356]. Inhibitors of Na-Pi-cotransporters or ATPase or reduced ATP bioavailability result in inhibition of bone Pi resorption [356], leading to increased plasma Pi levels. As previously described, F^−^ reduces ATP bioavailability and impairs glycolysis, and it is well established that F^−^ is an inhibitor of ATPase. Furthermore, recent in vivo studies with rodents and subsequent in vitro studies on bone tissues found that low dose F^−^ exposure stimulates expression of RANKL [357]. As previously elucidated, RANKL stimulates Pi release. Consistent with the mechanisms elucidated above, several human and animal studies have shown that F^−^ intake can enhance Pi levels [349,358,359,360,361,362,363,364,365,366,367]. As Pi has been found to inhibit NKA activity, this provides a further mechanistic pathway by which F^−^ inhibits NKA activity. Furthermore, as previously elucidated, F^−^ inhibition of enolase is also significantly stronger in the presence of Pi [278] and F^−^ inhibition of enolase has previously been found to inhibit NKA activity [136].

### 4.9. Dopamine Inhibits Na^+^-K^+^-ATPase Activity

It is known that dopamine (DA) inhibits NKA activity [231,368,369,370,371,372,373,374,375] though the molecular mechanisms of inhibition remain elusive. Furthermore, it has also been reported that PKC and cAMP signalling contribute to dopaminergic inhibition of NKA activity [376]. To understand how DA inhibits NKA activity, it is important to point out that DA has been found to directly stimulate cAMP [377,378,379]. Furthermore, Vortherms et al. reported that persistent activation of DA receptors results in a compensatory increase in cAMP accumulation [378]. As previously elucidated, the production of cAMP leads to a reduction in ATP, which is required for NKA enzyme activity.

Several experimental animal and in-vitro studies on tissues/cells have demonstrated that F^−^ stimulates DA release [227,380,381,382,383], although how this occurs is not well understood. However, other studies suggest that this may be due to the effect of F^−^ on hypothalamus function. It is well established that the hypothalamus regulates pituitary TSH secretion by releasing thyrotropin-releasing hormone (TRH) [384,385] and excess TSH is associated with iodine deficiency and hypothyroidism [110]. In addition to TRH induced release of TSH, TRH stimulates DA release [386,387,388]. Furthermore, it has also been observed that hypothyroidism increases DA receptor sensitivity by increasing receptor concentration [389]. As previously described, human studies have consistently found that F^−^ can stimulate TSH production in humans [140,255,269]. Thus, F^−^ must also induce TRH secretion. Furthermore, the stimulatory effect of F^−^ on DA release has been observed in animal studies with drinking water F^−^ levels of 1 mg/L [382]. Interestingly, in this study, the highest levels of DA release were observed at 5 mg/L F^−^ in water above which DA release was found to decrease. Consistent with this finding, it has reported that stimulant-induced increases in endogenous DA levels trigger feedback mechanisms that inhibit DA neuron firing [390]. Interestingly, inhibitors of NKA have also been found to almost completely abolish the TRH-induced DA releasing effect [378]. Therefore, it is also plausible that the reduction in DA release at higher F^−^ doses may reflect the enhanced inhibitory of F on NKA activity which in turn inhibits TRH induced DA release. It is also important to understand that biosynthesis of TRH in the hypothalamus is dependent on ATP [391]. Thus, increased TRH synthesis leads to a reduction in ATP bioavailability. As previously elucidated NKA function requires ATP. In addition to the animal studies which found that F^−^ induces DA release, evidence from human studies have found that the level of DA in foetal brain tissue is elevated in foetuses of F^−^-exposed mothers with dental fluorosis, compared to foetuses from mothers without dental fluorosis living in non-F^−^ endemic areas [392]. Collectively, these results provide strong evidence that the ability of F to upregulate TRH or TSH secretion may contribute to DA dysfunction leading to enhanced DA release which in turn may contribute to NKA inhibition by a mechanistic action that appears to involve cAMP production and decreased bioavailability of ATP.

### 4.10. Parathyroid Hormone Inhibits Na(+)-K(+)-ATPase Activity

One of the major regulators and inhibitors of proximal renal tubule NKA activity is parathyroid hormone (PTH) [374,393,394,395,396]. Further research has shown that PTH inhibits NKA through activation of PKC [393,397,398], cAMP [395] and phospholipase A2 (PLA2), pathways [397]. Furthermore, human studies have shown that when urinary F^−^ levels are in excess of 1 mg/L PTH expression is significantly enhanced compared to subjects with urinary F levels less than 0.5 mg/L [399]. It is important to point out that Schwartz et al. demonstrated that lowering of blood ionized calcium by an amount as low as 0.02 mmol/L (0.08 mg/L) within 30 min can elicit an immediate large, transient peak release of PTH amounting to 6–16 times the baseline concentration [400]. Of critical importance, Karademir et al. demonstrated that serum calcium levels were 0.075 mmol/L and 0.1 mmol/L lower among subjects with urinary F^−^ levels of 0.70 mg/L and 0.90 mg/L respectively, compared to controls with urinary F^−^ levels of 0.20 mg/L [260]. Taken together, these findings provide a basis for the hypothesis that F^−^- induced PTH release contributes to NKA inhibition by a mechanistic pathway that involves PKC, cAMP and PLA2. The association between F^−^ and PLA2 expression will be discussed in the following section.

### 4.11. Hyperglycaemia Inhibits Na^+^ K^+^ ATPase Activity via Activation of PGE2 Production

It is known that NKA activity is inhibited by elevated glucose concentrations although the mechanism of suppression remains largely unknown [401,402,403,404,405]. It has also been reported that inhibition of NKA activity by hyperglycaemia could be an important etiological factor of chronic complications in diabetic patients [406]. Importantly, a large number of human and animal studies have demonstrated that F exposure can induce hyperglycaemia [363,407,408,409,410,411,412,413,414,415,416,417,418,419,420]. Consistent with this, the U.S. National Research Council (NRC) reported that the conclusions from available studies is that sufficient F^−^ exposure appears to bring about increases in blood glucose or impaired glucose tolerance in some individuals and the increase the severity of some types of diabetes [150]. Again, it has been shown that mechanistic pathway by which hyperglycaemia inhibits NKA activity is via activation of PKC and phospholipase A2 (PLA2), resulting in the liberation of arachidonic acid (AA) and increased the production of prostaglandin E2 (PGE2), which are known inhibitors of NKA activity [406,421,422]. Importantly, several in vitro human tissue models have consistently demonstrated that F^−^ in micromolar concentrations of 1–10 µM significantly increases the synthesis of cAMP, AA, PGE2 and PLA2 in a dose dependent manner [249,423,424]. Taken together these observations provide a basis for the hypothesis that F^−^-induced hyperglycaemia contributes to NKA inhibition. Furthermore, evidence suggests that F^−^ may also inhibit NKA activity directly via stimulation of PLA2 synthesis, leading to increased production of AA and PGE2.

It is also important to point out that nuclear transcription factor kappa-B (NF-κB) has been previously reported to be involved in the up-regulation of COX-2 and generation of PGE2 [425,426]. It is well established that F^−^ activates the NF-κB mRNA expression in a wide variety of cell types and including monocytes, macrophages as well as lung, kidney and brain tissue [427,428,429,430,431,432,433,434]. This stimulatory effect has been observed at F^−^ concentrations of 2.5 µM [427]. However, the seminal study by Misra et al. demonstrated that beryllium fluorides at concentrations as low as 0.002 uM significantly upregulated the activation of NF-κB in macrophages [435], indicating that beryllium fluoride complexes have much greater cytotoxicity and genotoxicity than F^−^ alone. With an increasing interest in beryllium, concern has raised specifically about the risks of co-exposure to beryllium and F^−^ [435]. Human biomonitoring studies have determined that the mean concentration of beryllium in breast milk is 0.008 µg/L [436]. However, a UK study found that the mean concentration of beryllium in ten different brands of powdered infant formula products was 1.1 µg/L [437]. In view of the fact that infant formula products contain such high levels of beryllium and that beryllium is known to bind with high affinity to the electronegative F^−^ [438,439,440,441] raises particular concerns considering that infant formula products are reconstituted with fluoridated tap water in countries with water fluoridation.

### 4.12. Advanced Glycation end Products Inhibit Na^+^ K^+^ ATPase

Finally, it is known that the inhibition of enolase results in the formation of advanced glycation end products (AGEs) [442] and AGEs inhibit NKA enzyme activity [443]. Again, the mechanistic pathway has been elucidated to involve activation of AA metabolism via PLA2 activation [443]. It has been known for many decades that enolase is particularly sensitive to F^−^ inhibition [275,276,277]. Furthermore, recent in vivo rodent studies demonstrated that chronic long-term exposure for six months to F^−^ via drinking water significantly increased expression of receptors for advanced glycation end products (RAGE), increased RAGE proteins and increased levels of AGEs in cells. A significant increase in the expression NADPH oxidase 2 (NOX2) was also observed among specimens exposed to fluorine for 6 months. Notably these effects were found to occur at concentrations of just 5 mg/L in drinking water, which is the equivalent to approximately 0.5 mg/L in drinking water for humans. Simultaneous in vitro research with SH-SY5Y cells originating from human neuroblastoma confirmed these results [444]. Taken together these observations provide a basis for the hypothesis that F^−^-induced inhibition of enolase contributes to NKA inhibition by activation of AGEs, which in turn leads to activation of PLA2 and the synthesis of AA which is metabolised to PGE2.

## 5. Discussion

As noted from the preceding data, F^−^ inhibition of NKA activity is complex and multifactorial. In summary, evidence is provided to show that activation of PKC, cAMP, cGMP, NO, Pi, PLA2, AA and PGE2 inhibit NKA activity and that F^−^ upregulates PKC, cAMP, cGMP, NO, PLA2, AA, PGE2 and enhances Pi levels in systemic circulation. Furthermore, evidence is presented to show that Cn regulates NKA activity and that F^−^ inhibits CN activity, in part, by regulating the bioavailability of manganese and Pi, as well as by altering the phosphorylation activity of CaM. In addition, evidence is provided to show that F^−^ enhances the activation of adenylate cyclase by CaM, leading to increased levels of cAMP and further evidence is provided to show that F^−^ inhibits the activation of phosphodiesterase by CaM, which is responsible for the breakdown of cAMP. I further elucidate that the presence of magnesium facilitates the binding of ATP to NKA, thereby providing the chemical energy required for ion channel function and secondary active transport. I have described how F^−^ contributes to magnesium deficiency by forming complexes with magnesium thereby lowering the absorption of magnesium and F^−^ in the gastrointestinal tract leading to reduced bioavailability. I have further elucidated that F^−^ retards the mobilization of magnesium in bone and can increase the excretion of magnesium in urine bound to ALB.

In the present study, I have further elucidated the molecular mechanisms by which hypothyroidism lowers NKA activity. In addition, I describe how F^−^ inhibition of enolase leads to inhibition of glycolysis thereby reducing ATP production. As ATP is required for NKA function, lower levels of ATP in turn may lead to inhibition of enzyme activity. Furthermore, evidence is presented to show that NKA activity is inhibited by cAMP and that F^−^ acts to induce cAMP production. Moreover, in this study I have elucidated that there are two distinct mechanisms by which cAMP inhibits NKA activity. This can occur through loss of ATP, as well as the direct effect of cAMP in enhancing phosphorylation of the NKA α subunit. In this study, I also describe how the pituitary hormone TSH increases cAMP production and further evidence is provided to show that F^−^ upregulates TSH secretion leading to a positive feedback mechanism that may result in inhibition of enzyme activity. I have further described how Pi has been found to inhibit NKA activity. I have further elucidated the role of ALP and RANKL in Pi release and the contribution of F^−^ to increased mRNA expression of RANKL and ALP activity, which contributes to increased concentration of Pi in serum. Moreover, I have described how ALP activity is increased in the presence of CT and that F^−^ is a potent inducer of CT activity. I have also described how CT stimulates TGF-β1 protein synthesis as well as TGF-β1 mRNA expression and that TGF-β1 inhibits the expression of the β-subunit of NKA required for enzyme function.

Further evidence is presented to show that NKA activity is inhibited by dopamine, PTH and glucose. In addition, evidence is presented demonstrating that F^−^ upregulates dopamine, glucose and PTH activity. Further analysis reveals that the mechanism of dopamine inhibition of NKA is via increased production of cAMP. It has also been elucidated in this study that NKA inhibition by PTH and hyperglycaemia is regulated by activation of PLA2, AA and PGE2. In addition, there is evidence demonstrating that stimulation of AGEs inhibits NKA activity and that F^−^ inhibition of enolase results in the formation of AGEs. Again, the mechanistic pathway by which AGEs inhibit NKA enzyme activity is via activation of the PLA2 pathway leading to enhanced expression of AA and PGE2. I have also elucidated how F^−^ induces NF-κB mRNA expression in a wide variety of cell types and how NF-κB activation leads to increased PGE2 production. Consistent with these findings, evidence is provided from several in vitro studies with human cell lines that have consistently demonstrated that F^−^ at biologically relevant exposures, ranging from 1–10 µM, increases the synthesis of cAMP, PLA2, AA and PGE2 in a dose dependent manner. Moreover, evidence from human studies confirm that F^−^ exposure as measured by serum F^−^ levels in adults, inhibits NKA activity in vivo at levels within the same range observed in human cell in-vitro studies showing increased activation of cAMP, PLA2, AA and PGE2. While this effect on NKA was found to occur in adults at serum F^−^ levels < 5.0 µM, at higher mean serum F^−^ levels of 14.75 µM the activity of NKA declined by approximately 60 per cent compared to controls [137,138]. Moreover, the serum F^−^ levels observed in the control groups of either of these two studies were comparable to the mean fasting serum F^−^ concentrations recently reported among healthy adults of similar age residing in a non-fluoridated community (drinking water F^−^ level < 0.3 mg/L) in the UK [188]. This data suggests that total F^−^ intake among adults in western countries where drinking water is fluoridated or where habitual tea drinking is commonplace and where F^−^ toothpaste is widely available would fall within the range observed to cause inhibition of NKA. Furthermore, it is important to acknowledge that in addition to inhibiting NKA activity, Arulkumar et al. also found that the activity of adenosine 5′ triphosphatase (ATPases), Mg(2+) ATPases, acetylcholinesterase (AChE), butyrylcholinesterase (BChE), paraoxonase 1 (PON1) and arylesterase (ARE) declined in a dose dependent manner with increasing serum F^−^ concentrations [137]. Clearly, further studies are warranted to explain the wider implications associated with F^−^ inhibition of these important enzymes. For example, loss of ARE and PON1 is associated with metabolic syndrome and are considered independent risk factors for cardiovascular disease [445]. Moreover, downregulation of AChE causes inflammatory hyperactivation of the CNS and peripheral nervous system [446,447]. Moreover, the U.S.A. National Academy of Sciences NRC previously reported that F^−^ downregulates AChE and suggested that this action may contribute to increases the risk of developing Alzheimer’s disease [150].

Returning to the hypothesis/question posed at the beginning of this study, it is important to assess the implications of F^−^ inhibition of NKA activity on human health and disease inequalities. As I have described, loss of NKA activity has been implicated in many pathophysiological conditions, including asthma and allergic diseases, metabolic disorders, cancer, cardiovascular disease, as well as neurodevelopmental and degenerative brain diseases. Past studies have further suggested that inhibitors of NKA may also contribute to disorders associated with loss of NKA activity [18]. This hypothesis is supported by two current studies which infer a causal association between F^−^ exposure and pathophysiological states associated with loss of NKA activity including iodine deficiency [110] and degenerative eye diseases [53]. Among the many molecular mechanisms identified in these latter studies was the contribution of F^−^ to loss of NKA activity.

As I already described, loss of NKA activity has been implicated in asthma [19,20], and allergic diseases such as allergic rhinitis [22,23,24]. Moreover, the seminal study by Gentile et al. provided both correlative and mechanistic evidence for a causal relationship between NKA enzyme inhibition and airway hyperreactivity (or bronchial hyperresponsiveness) among asthmatic and allergic subjects [18]. Furthermore, Gentile et al. concluded that inhibitors of NKA could play a role in the pathogenesis of AHR in human beings. Indeed, this observation has already been reported in several studies including environmental and occupational exposure studies. For example, Søyseth et al. found that exposure to atmospheric fluorides corresponded to an increase bronchial hyperresponsiveness in children aged 7–13 years [448]. As described in this current study, F^−^ has consistently been found to inhibit NKA activity. Therefore, evidence suggests that F^−^ may also play a role in the pathogenesis of bronchial hyperresponsiveness and inflammatory respiratory diseases. Moreover, earlier occupational epidemiological work by Søyseth et al. suggested that F^−^ exposure was likely to be a causative agent in causing asthmatic symptoms among workers in the aluminium smelting industry [449]. Of fundamental importance, a positive dose-response association was observed between bronchial responsiveness and plasma F^−^ levels, such that an increase in the plasma F^−^ level of 0.5 µM was associated with an increase in the dose–response slope by a factor of 1.11 (95% CI, 1.05 to 1.17). Furthermore, the authors hypothesized that an increase of plasma F^−^ of 3.4 µM would be associated with a doubling of bronchial responsiveness [449]. The association between bronchial responsiveness and elevated plasma F^−^ is important, as several studies have found that increased bronchial responsiveness is associated with asthma [450,451,452,453,454] and impaired lung function [455,456,457].

This leads us to perhaps the most significant evidence revealed in this current study regarding postnatal and early infant chronic F^−^ exposure which has been found to occur in communities with artificially fluoridated drinking water (AFDW). As previously elucidated the plasma F^−^ levels in infants residing in communities with optimally fluoridated water can be extremely high, due to the reconstitution of powdered infant formula with fluoridated tap water. As described, past research has shown that the mean plasma F^−^ levels in infants residing in a community with AFDW during their first 18 months of life was 3.16 μM, with significantly higher plasma F^−^ levels measured in infants aged 4–6 months (mean 4.22 µM with a maximum of ~ 8 µM). I have previously elucidated that this level of exposure is comparable to that observed among adult workers occupationally exposed to F^−^ in the aluminium industry and within the range observed in human studies associated with endemic fluorosis. Importantly, the plasma F^−^ levels are also within the reported range in human studies to cause inhibition of NKA activity in adult subjects.

However, it is not enough to simply extrapolate from research among adults. As previously described, infants have a lower low glomerular filtration rate which results in higher retention of F^−^. The infant also has an immature blood-brain barrier [458,459] and neonates and infants have lower antioxidant activities than adults [460,461]. Moreover, as previously described, infants can be exposed to beryllium fluoride complexes from the consumption of powdered in formula reconstituted with fluoridated tap water. The interaction of beryllium with F^−^ is critical, as it has been shown that beryllium F^−^ complexes are significantly more toxic than F^−^ or beryllium alone, resulting in significantly increased production of NF-κB, which leads to increased production of PGE2. As previously elucidated PGE2 is known to inhibit NKA activity. Therefore, it is plausible that the inhibitory effects of chronic F^−^ exposure on NKA activity may be higher in neonates and infants that adults, particularly in communities where drinking water is artificially fluoridated or where F^−^ levels in drinking water are naturally elevated. Because newborns and infants are the group most different anatomically and physiologically from adults, they exhibit the most pronounced quantitative differences in sensitivity to chemical exposures. For this reason, the U.S.A. National Research Council [462] and others [463] propose using a 10-fold factor when extrapolating results from studies using adult exposures when estimating safe exposures to chemical toxins for the protection of infants. Given that this exposure has been found to occur at such a sensitive period of development and that loss of NKA activity represents an interconnected molecular function in neurodevelopmental, neuropsychiatric and neurodegenerative disorders, which is also connected with other pathophysiological states, this suggests the possibility that chronic F^−^ exposure in infancy could have profound implications for neurobehavioral function and later health. Indeed, it would be naive to assume that such exposure during this critical period of development is without negative consequence to long term health. Clearly, further studies are warranted to explore these relationships. Failure to do so leads to conclusions and recommendations regarding water fluoridation that are not reliable, and therefore public health practices that are not reliably safe and effective. In addition, the fact that F has been found to activate NF-κB mRNA expression in a variety of cells types including brain, lung and kidney tissue is highly relevant. As previously described NF-κB increases production of PGE2, and PGE2 inhibits NKA activity. This elucidation may explain why NF-κB activation has been linked to many neurological disorders including autism [464,465,466,467], Alzheimer’s disease [468,469,470,471], Parkinson’s disease [472,473], as well as asthma, COPD, diabetes and cancer [474,475].

Furthermore, in view of my demonstration that F^−^ exposure and loss of NKA plays a role in inflammatory lung diseases, this suggests that the significantly higher burden of childhood respiratory disorders documented in Australia, New Zealand, the Republic of Ireland (RoI) and North America compared to other developed peer countries without AFDW, as noted in large scale epidemiological studies [476,477,478,479], may be causally associated with chronic F^−^ exposure in infancy and F^−^-induced inhibition of NKA. It is important to note that in Australia, just 15% of infants are fully breastfed to six months of age [480] with 80% of infants provided with powdered infant formula at 6 months of age and 95% at 12 months [481]. Moreover, the RoI has the lowest prevalence of breastfeeding internationally [482]. A recent Irish birth cohort study found that only 14% of babies were exclusively breastfed at two months of age and just 1% at six months [483]. Similar low breastfeeding prevalence rates to Ireland have been reported among Maori and Pacific Island women in New Zealand [484] and among mothers from lower socio-economic backgrounds in the United States [485]. In 2009, Siew et al. measured the F concentrations in 21 milk based powdered infant formula products available in the United States and reported that the F levels ranged from 0.03–0.27 ppm when prepared with deionized distilled water. However, twenty-five per cent of the products were found to contain F levels ranging from 0.22–0.27 ppm F [161]. Any of these products when reconstituted with AFDW would result in F levels exceeding the UL for healthy adults. It is also important to note that in countries where AFDW is widely available the prevalence of childhood asthma has increased dramatically and disproportionally to other peer countries in recent decades and this increase parallels the increased prevalence of dental fluorosis. For example, in late 1960s and 1970s asthma prevalence among children in Australia [486] and the USA [487] was less than 4%. Similarly, in 1983, asthma prevalence among children aged 4–19 years of age was 4.4% in the RoI [488]. In contrast, Masoli et al. reported that the prevalence of current asthma symptoms among children aged 13–14 years in the USA, RoI, Australia and New Zealand were 21%, 28%, 30% and 32% respectively in 2004 [489]. Today, the burden of asthma in countries with water fluoridation is of sufficient magnitude to warrant its recognition as a priority disorder in government health strategies. As mentioned, these dramatic changes mirror almost exactly the changes in prevalence of dental fluorosis which occurred during the same period. To illustrate this point, in the RoI, Whelton et al. reported that in 1984 the prevalence of dental fluorosis among 8 and 15-year olds in the RoI was 6% and 5% respectively, increasing to 23% and 36% in 2002 [490]. Similarly, in the US the prevalence of dental fluorosis was 9% among individuals born in the period 1961–1970, compared to 41% among all US children born between 1984–1985 [491]. In Australia, the prevalence of dental fluorosis among children born in 1989/90 was reported to be 34.7% [492]. Interestingly, breastfeeding practices in France are among the lowest in Europe and lower than North America, Australia and New Zealand [482]. In comparison to the United States, RoI and Australia, a study conducted in France in 1998 reported that 97% of children had no sign of dental fluorosis, and 3% mild, very mild or doubtful fluorosis without aesthetic consequences [493]. A similar study conducted in Germany in 2007, reported that the prevalence of dental fluorosis among children aged 15 years ranged from 7.1% to 11.3% [494]. Notably, childhood asthma prevalence in Europe, including Germany and France is significantly lower than Australia, New Zealand, RoI and North America [476,477,478,479]. Despite these obvious associations, no study has ever been conducted to examine the causal association between chronic F intake in infancy and childhood asthma. Clearly, given the burden of childhood asthma in countries with AFDW such studies are warranted.

As previously discussed, I have also elucidated that maternal hypothyroidism in pregnancy can results in loss of NKA activity in offspring that leads to marked reduction in enzyme activity in later life. Animal models of F-induced hypothyroidism have also shown that excessive intake of F in drinking water and prenatal F intoxication of mothers induces hypothyroidism in offspring [495,496,497]. Interestingly, a recent animal study also found that maternal exposure to F during pregnancy and early postnatal life exposure had deleterious impact on learning and memory of offspring which was mediated by reduced mRNA expression of glutamate receptor subunits in the hippocampus [498]. Furthermore, the inhibition of glutamate receptors by F was found to occur in a dose dependent manner. Clearly, inhibition of mRNA expression of glutamate receptors can lead to a loss of glutamate receptors. Loss of glutamate receptors can subsequently lead to excessive activation due to their impaired expression, which can lead to enhanced excitotoxicity from chronic glutamate toxicity. These elucidations strongly suggest that chronic F exposure can attenuate adverse effects associated with glutamate excitotoxicity. Consistent with this hypothesis, an earlier study by Blaylock suggested that F exposure may contribute to glutamate induced excitotoxicity, thought the effects of F on mRNA expression of glutamate receptors were not known at that time [499]. While these observations may not be part of the original goal of this study, they are clearly important because, as previously discussed, loss of NKA has been suggested to enhance glutamate excitotoxicity [95,96] and glutamate excitotoxicity is associated with major psychiatric disorders [98,99,100,101], neurodegenerative diseases [102] and autism [103,104]. Furthermore, it is also important to note, that in addition to inhibition of glutamate receptors, it has also been found that maternal exposure to F during pregnancy results in inhibition of mRNA levels of M1 and M3-muscarinic acetylcholine receptors (mAChRs) in offspring [500]. Similar results have been observed in adult rodents chronically exposed to F in drinking water [501]. Interestingly, in addition to loss of NKA activity, a reduction or deficiency in mAChR have also been implicated in the pathophysiology of many major diseases of the CNS including schizophrenia [502], bipolar and major depression [503], Alzheimer’s disease [504] and ADHD [505]. Loss of M3 mAChRs has also been found to result in impairments in glucose tolerance and insulin release [506]. Taken together, these results further strengthen the hypothesis that F exposure can contribute to etiology and pathophysiology of a diverse range of disorders.

Furthermore, in this study I have described how F exposure can result in increased TSH and higher TSH is associated with iodine deficiency and hypothyroidism. Consistent with these findings, I have discussed how evidence from human studies indicate that water fluoridation is associated with increased prevalence of hypothyroidism [139]. Moreover, it is well acknowledged that iodine deficiency and maternal hypothyroidism is associated with increased risk of cognitive impairment in offspring [507,508,509], along with increased risk of ASD and ADHD [510,511,512,513,514,515], schizophrenia [516], epilepsy and seizures [517]; and asthma [518]. As elucidated in this study, loss of NKA activity has been found to play a central role in these disorders. This evidence further supports the hypothesis that prenatal loss of NKA is implicated in disorders associated with maternal hypothyroidism. Taken together, these findings suggest the possibility that paternal exposure to F can have epigenetic transgenerational effects on future generations. Indeed, this observation has already been observed in several rodent studies [519,520,521,522]. It is not known however, whether inhibition of NKA activity during the early postnatal period and early infancy can persist during the entire lifespan. Nonetheless, this possibility clearly exists, as several studies have found that early life exposure to environmental chemicals and stress can result in epigenetic changes by reprogramming gene expression patterns, which persist into adulthood [523,524,525,526,527]. Clearly, further research is warranted to elucidate whether chronic F exposure in early infancy results in epigenetic changes in gene expression. This is particularly important given the seminal research of Liu et al. where they found that chronic F exposure resulting in dental fluorosis, altered the expression of over 960 genes in children compared to controls without dental fluorosis, including 71 robustly up-regulated genes and 60 robustly down-regulated genes [528].

Moreover, in this study I have elucidated that chronic F exposure has been found to inhibit the expression of PERK, which is required to stimulate WFS1 expression. I have further described how loss of WFS1 expression leads to reduced expression of the NKA β sub unit which is required for expression of NKA on plasma membranes and for enzyme activity. Thus, F inhibition of PERK can lead to reduced NKA expression and lower enzyme activity. Interestingly, loss of WFS1 activity is also associated with increased risk of psychiatric disorders [529,530,531], as well as juvenile-onset diabetes, progressive neurologic degeneration, and endocrine dysfunction [532,533]. The association between loss of WFS1 and juvenile diabetes is particularly interesting considering the dramatic increase in juvenile diabetes in the United States in recent decades [534], which also happens to coincide with the dramatic rise in dental fluorosis observed in the United States in recent decades [535,536]. Moreover, it should be noted that an association has been found between drinking water F^−^ levels and incidence of childhood-onset type 1 diabetes has been observed in Canada [537]. Furthermore, a recent ecological study in the USA reported an association between water fluoridation and diabetes [538]. Revealingly, studies have also shown that PERK protects pancreatic β-cells from ER stress [532] and PERK deficiency is associated with hyperglycaemia and increased apoptosis in β-cells [539]. Taken together, these observations may explain why loss of NKA activity has been found to be associated with psychiatric disorders, metabolic syndrome and diabetes. They also provide insights into molecular mechanisms by which chronic F^−^ exposure may contribute to these disorders.

In this present study, I have also elucidated that evidence from human studies implicate loss of NKA with the pathogenesis of COPD [21]. According to WHO, COPD will move from fifth leading cause of death in 2002, to fourth place in the rank projected to 2030 worldwide [540]. Notably, in the USA, death rates for COPD doubled between 1970 and 2002 [541]. It is also evident that the RoI, New Zealand and Australia, have by far the highest prevalence rates for COPD among developed countries despite having an adult smoking prevalence well below the OECD average [542]. Interestingly, Australia, New Zealand and the RoI, also have the highest age-standardised incidence rates of cancer worldwide for men and women together being ranked 1st, 2nd and 3rd, with the USA in 5th place [543,544]. As previously described, several studies have also found that loss of NKA is implicated with carcinoma and cancer progression [54,55,56,57,58,59,60]. Since loss of NKA has been found to be associated with both cancer risk and inflammatory respiratory lung diseases, this suggests a plausible scenario that F^−^ intake may be a contributory factor to the high burden of COPD and cancer in countries with artificial water fluoridation. While these associations are self-evident, they are merely observations and do not prove causality, nevertheless a causal biological mechanism exists, making the hypotheses plausible. Clearly, additional studies in this important area of investigation are also warranted.

Past studies have also shown that reduced NKA activity may underlie the pathophysiological aspects linked to the prehypertensive status in humans [40,545]. Additionally, it has been widely documented that inhibition of NKA activity is associated with hypertension [44,45,46,47,48,49,50,51,52,53]. Again, several human studies have found that exposure to excessive F is closely associated with hypertension [546,547,548,549,550]. Similar observations have been observed in experimental studies with rodents [551,552,553,554,555,556]. Interestingly, activation of the TRH system, with increased production of TRH and an upregulation of its receptors has also been implicated in the pathogenesis of hypertension [557]. As previously, described, TRH regulates the secretion of TSH and stimulates the secretion of DA, which can lead to inhibition of NKA activity. As previously described, evidence from human studies have shown that F^−^ can induce TSH secretion. Therefore, F^−^ must also induce TRH secretion. This elucidation may explain why increased TRH release is associated with lower NKA activity. Moreover, these findings are consistent with the hypothesis that F exposure contributes to pathological states associated with loss of NKA activity.

Furthermore, in this current study, I have provided compelling evidence that NKA activity is vital for normal brain development and loss of NKA activity is associated with cognitive impairment, neurological and developmental disorders [14,15,62,63,64,65,66,67,68,69,70,71,72,73,74,75,76,77,78,79,80,81,82,83,84,85,86]. Indeed, loss of NKA activity represents an interconnected molecular function in neurodevelopmental and neuropsychiatric and neurodegenerative disorders including; Down syndrome, Alzheimer’s, Parkinson’s and Huntington’s disease, as well as epilepsy, autism, schizophrenia, mood and depressive disorders. Furthermore, evidence has been presented that loss of NKA activity is also associated with allergic diseases, blood diseases, autoimmune diseases, metabolic disorders, male infertility and cardiovascular disease. Therefore, it is plausible that F^−^-induced loss of NKA activity could lead to pathological states or further contribute to the severity of a diverse range of inflammatory diseases/disorders associated with loss of enzyme activity. Of particular note, while loss of NKA activity has been shown to be associated with autism spectrum disorder’s, research has also shown that inhibition of NKA activity can ultimately lead to a leaky and dysfunctional epithelium associated with chronic inflammation [558]. Accumulating evidence demonstrates that gastrointestinal inflammation and increased permeability of the intestinal tract, referred to as a “leaky gut” is a hallmark of ASD and the severity of gastrointestinal symptoms relate to the severity of ASD [559]. This suggests that a potential adverse effect of chronic F^−^ exposure may include contributing to the burden and severity of ASD. In this study, I have also elucidated how loss of NKA leads to downregulation of AMPA receptor, which leads to synaptic transmission defects, and consequently cognitive impairment [97]. I have further described how loss of AMPA receptors have been found to result in early-onset motor deficits, hyperactivity, cognitive defects and behavioural seizures [105]. I have further elucidated that these findings suggest a possible causal mechanism explaining how loss of NKA activity is associated with childhood neurodevelopmental disorders such as ADHD and ASD. Consistent with this, several studies have already demonstrated an association between cognitive impairment and loss of NKA activity and between loss of NKA and ASD. Importantly, these findings may also explain why exposure to fluoridated water has been found to be associated with increased prevalence of ADHD in the USA [560]. Interestingly, one of the most common difficulties in children with epilepsy is ADHD. Indeed, in children with epilepsy (seizures), ADHD has been found to be present in 20–50% of patients [561]. These findings further support the hypothesis that downregulation of AMPA receptors which results from loss of NKA activity is a factor in the pathogenesis of ADHD disorders. Furthermore, these findings may elucidate a key mechanism by which prenatal exposure to F^−^ has recently been found to be associated with cognitive impairment and increased risk of ADHD in offspring [562,563].

Increasing evidence also suggests that maternal iodine deficiency and hypothyroidism is associated with increased risk of cognitive impairment, neurodevelopmental and neuropsychiatric disorders in offspring including ADHD [511,512,513,514,515,516,517,518,519,520,521,522,523,524,525,526,527,528,529,530,531,532,533,534,535,536,537,538,539,540,541,542,543,544,545,546,547,548,549,550,551,552,553,554,555,556,557,558,559,560,561,562,563,564,565], ASD [566,567,568], behavioural seizures [517] and schizophrenia [516]. Moreover, maternal iodine deficiency and hypothyroidism is also associated with increased risk of asthma [518] and hypertension [569] in offspring. As elucidated in this study, loss of NKA activity appears to be a critical contributor to the pathophysiological underpinnings of these disorders. These findings suggest that loss of NKA activity is associated with iodine deficiency disorders. Consistent with this, I have previously elucidated that NKA is essential for NIS functionality, iodine uptake and metabolism. Indeed, I recently reported that inhibition of NKA contributes to iodine deficiency disorders [110]. As highlighted above, there is also an association between hypothyroidism and loss of NKA activity, suggesting a negative feedback mechanism that may further decrease enzyme activity. As elucidated in this current study, this mechanism appears to be driven by increased TRH secretion and DA release which leads to inhibition of enzyme activity.

### Additional Perspectives

Reflecting on the established link between loss of NKA activity and increased risk of cancer, metabolic, pulmonary and cardiovascular disease as well as neuropsychiatric and neurodegenerative disorders, the indisputable fact that F^−^ has consistently been found to inhibit NKA activity suggests the possibility that populations with increased F^−^ intake may be susceptible to diseases associated with loss of NKA activity. In considering this hypothesis, the population on the island of Ireland offers an ideal model system for investigating the relationship between F^−^ exposure and adverse health effects associated with loss of NKA activity.

As previously discussed, drinking water is artificially fluoridated in the Republic of Ireland (RoI) and non-fluoridated in Northern Ireland (NI). The island of Ireland consists of an area of 84,421 km^2^ of which the RoI covers five sixths of the island or 70,273 km^2^, with NI constituting the remainder. The similarities in populations are reflected in 2011 census reports, which showed that the majority of people living on the island of Ireland were of a White ethnic background, which accounted for 98 per cent of those usually resident in Northern Ireland and 94 per cent of those in the Republic of Ireland [570]. Moreover, the median age of the population in the RoI is 34, compared to 37 in NI. The rates of young, working age and older age dependency for NI and the RoI are also similar at 52 and 49 per cent respectively. In addition, working age groups (aged between 19 and 64 years) made up 60 per cent of the population in NI, compared with 62 per cent in the RoI [570].

It is therefore pertinent to note that there is now considerable direct evidence derived from epidemiological studies that cancer incidence and mortality from inflammatory diseases is notably higher in the RoI than in NI. Notably, a previous study by the Institute of Public Health in Ireland examined mortality from leading causes of death for the whole island, NI and the RoI inclusive between 1989 and 1998 [571]. While the report recommends caution in the interpretation of findings highly significant difference were observed. For example, the directly standardised mortality rate (DSRRs) for diabetes mellitus was 371% higher in the RoI compared to NI. In addition, significant differences in the DSRRs were observed for endocrine, nutritional and metabolic diseases (245% higher); sudden infant death syndrome (210% higher); rheumatoid arthritis and osteoarthrosis (166%); diseases of the blood and blood-forming organs, immunological disorders (148% higher); heart diseases including acute pericarditis, acute and subacute endocarditis, acute myocarditis, cardiomyopathy, conduction disorders, cardiac dysrhythmias and heart failure (88% higher); mental and behavioural disorders (53% higher), chronic lower respiratory disease (44% higher), asthma (34%), diseases of the kidney and ureter (27% higher); diseases of the skin and subcutaneous tissue (26% higher) and cancer, including malignant neoplasms of the ovary (23% higher), prostate (19% higher); pancreas (18% higher); uterus (18% higher); skin (16% higher); oesophagus (15% higher); lymph/haematopoietic tissue (12% higher); colon (10% higher); breast (8% higher); cervix uteri (6% higher); and stomach (4% higher) [571].

More, recently, the National Cancer Registry Ireland and the Northern Ireland Cancer Registry published the findings of the All-Ireland cancer incidence for the period 1994 to 2004 [572]. In this report, statistically significant differences in incidence rates (EASIR) were found to exist with significantly lower incidence rates for cancer in NI compared to the RoI. Incidence rates in RoI were higher for cancer of the pancreas, bladder, brain, colorectal, prostate, cervical, breast, stomach, skin, oesophagus kidney and leukaemia [572]. A subsequent publication on cancer data from the island of Ireland, examining cancer incidence in NI and RoI provided similar disturbing findings [573]. According to the All-Ireland Cancer Registry (2011) the risk of developing prostate, bladder, pancreatic, oesophageal, ovarian, cervical, blood and bone cancers, non-melanoma and melanoma skin cancers and brain/central nervous system cancers was significantly higher in RoI compared to Northern Ireland [573]. Taken together these studies provide compelling evidence linking diseases associated with loss of NKA activity to the increased exposure of the population of the RoI to F^−^ from AFDW. Moreover, the observations from these studies reveal astonishing similarity to the findings of Takahashi et al. [574]. In this ecological study, the prevalence and geographic variation of a large number of cancers in the United States were found to be associated with water fluoridation. It is also pertinent to note that that the RoI has one of the highest rates of mental health illness in Europe with almost one in four of the population recorded as having a mental health disorder with a total cost to the Irish economy over €8.2 billion a year [575]. Interestingly, it has previously been reported that among the general population in the RoI the prevalence of schizophrenia is among the highest in the world [576,577]. It has recently been reported that among general practitioners in the RoI, seventy-three percent have between one and ten patients with schizophrenia on their list, and a further 27% have over ten [578]. According to the Central Statistics Office, the both men and women the highest cause of admission to psychiatric units in the RoI is for depressive disorders followed by schizophrenia [579]. It is also important to note that the United States has one of the highest prevalence’s of schizophrenia in the world with prevalence’s rates several fold higher than other developed countries [580]. Interestingly, Golder et al. also reported that the lowest prevalence reported in literature was found in Christchurch, New Zealand [580]. Notable, Christchurch, New Zealand is the largest urban population center in New Zealand where drinking water is non fluoridated. As previously elucidated loss of NKA is associated with schizophrenia, depressive and bipolar disorders. Taken together, the advances in understanding the molecular mechanisms by which F^−^ inhibits NKA activity and the role of loss of NKA activity in the etiology and pathophysiology of disease provides a plausible explanation for elucidating the reasons driving health inequalities in the island of Ireland and other countries with water fluoridation policies. Clearly, these associations are significant enough to warrant further research.

In this study I have elucidated that dephosphorylation of NKA is mediated by Cn and that inhibition of Cn activity leads to enhanced phosphorylation, which in turn leads to inhibition of enzyme activity. I have further described how F^−^ inhibits CN activity and elucidated the molecular mechanisms of inhibition including the contributory effect of F^−^ reducing manganese bioavailability. In addition, I have described how manganese is required for structural stability and full activity of Cn in addition to manganese being a crucial activator of Cn. To further our understanding of the molecular mechanisms by which F^−^ can induce neurotoxicity it is important to note that Cn is also thought to play a role in aspects of learning and memory by regulating synaptic plasticity and suppression of long-term depression (LPD) in the hippocampus [581,582] Studies have also found that inhibition of Cn causes impaired working memory [583] and inhibition of Cn has been implicated in the pathogenesis of Alzheimer’s disease [584]. Moreover, it has been shown that a reduction in function of Cn can lead to a spectrum of abnormalities related to schizophrenia, including defects in working/episodic like memory, hyperactive movement, social withdrawal, and defects in latent and pre-pulse inhibition in mice [585,586]. Of note, these abnormalities are also associated with autism spectrum disorder (ASD) and attention-deficit/hyperactivity disorder (ADHD) [587,588,589,590,591,592]. Based on these findings, further studies are also warranted to provide objective evidence to evaluate adequately the association between F^−^ exposure, Cn expression, NKA activity and cognitive impairment, Alzheimer’s disease, schizophrenia, autism and ADHD.

In this study, I have also elucidated how F^−^ contributes to magnesium deficiency. It is important to note that magnesium deficiency is highly prevalent in many countries including the United States. where up to thirty per cent of the population have been reported to have subclinical magnesium deficiency, based on serum magnesium levels [591]. A previous study reported that among hospitalised patients in the United States the prevalence of hypomagnesaemia was identified in 47% of patients [592]. Although low magnesium status may not be a direct cause of inflammatory diseases, insufficient magnesium has consistently been shown across multiple laboratories to increase chronic low-grade inflammation, which is thought to play a major role in chronic disease etiology.

Furthermore, a growing body of literature from animal, epidemiologic, and clinical studies has demonstrated a varied pathologic role for magnesium deficiency that includes electrolyte, neurologic, musculoskeletal, and inflammatory disorders; osteoporosis; hypertension; cardio-vascular diseases; metabolic syndrome; and diabetes [591]. As described in this study magnesium is required for ATP binding to NKA, thus, magnesium deficiency contributes to lower NKA activity. This elucidation may in part explain why magnesium deficiency is associated with pathologic conditions of the human nervous, metabolic, cardiovascular and muscular system that are also associated with loss of NKA activity.

## 6. Conclusions

In this study, several lines of evidence are provided to show that NKA activity exerts vital roles in normal brain development and function and that loss of enzyme activity is implicated in neurodevelopmental, neuropsychiatric and neurodegenerative disorders, as well as increased risk of cancer, metabolic, pulmonary and cardiovascular disease. Evidence is presented to show that F^−^ inhibits NKA activity by altering biological pathways through modifying the expression of genes, glycolytic enzymes, hormones, proteins, neuropeptides and cytokines, as well as biological interface interactions that rely on the bioavailability of chemical elements magnesium and manganese to modulate ATP and NKA enzyme activity resulting in loss of enzyme activity. Further, evidence from human studies show that inhibition of NKA activity occurs at biological relevant doses and the inhibitory effect increases as serum F^−^ levels rise in a dose dependent manner. Taken together, the findings of this study provide unprecedented insights into the molecular mechanisms and biological pathways by which F^−^ inhibits NKA activity and contributes to the etiology and pathophysiology of diseases associated with impairment of this essential enzyme activity. Moreover, the findings of this study further suggest that there are windows of susceptibility over the life course where chronic F^−^ exposure in pregnancy and early infancy may influence NKA activity with both short- and long-term implications for disease and inequalities in health. These findings would warrant considerable attention and potential intervention, not to mention additional research on the potential effects of F intake in contributing to chronic disease.

## Figures and Tables

**Table 2:** Key molecular mechanisms by which fluoride inhibits Na^+^, K^+^-ATPase activity. Arrows refer to increases (↑) or decreases (↓) in regulation or expression by fluoride.

Factor	Effect of F^−^	Effect on Na^+^, K^+^-ATPase Activity
ATP	↓	ATP is required for NKA homeostasis. Lower bioavailability of ATP leads to inhibition of enzyme activity
ENO1	↓	Enolase is necessary for glycolysis and ATP production. Inhibition of enolase leads to loss of NKA activity
PKC	↑	PKC phosphorylates the α-1 subunit of NKA leading to inhibition of activity.
cAMP	↑	Inhibits NKA activity by decreasing bioavailability of ATP and enhancing phosphorylation of the α-1 subunit of NKA
Cn	↓	Regulates the dephosphorylation of NKA. Phosphorylation of NKA inhibits enzyme activity. Hence, inhibition or activation of Cn regulates enzymatic activity. Requires Calmodulin and Manganese for structural stability and full activity.
CaM	↑	Inhibits Na^+^, K^+^-ATPase activity by enhancing phosphorylation
Mn2+	↓	Mn2+ is also an activator of Cn and its binding to Cn is required for functional stability and enzyme activity. Loss of Mn^2+^ inhibits Cn expression and impairs Cn activity leading to enhanced phosphorylation of NKA. Phosphorylation inhibits NKA activity.
Mg2+	↓	Mg2+ facilitates the binding of ATP to NKA thereby providing the chemical energy required for enzyme activity.
cGMP	↑	Inhibits NKA activity
NO	↑	Inhibits NKA activity
Pi	↑	Inhibits NKA activity directly as well as inhibiting the phosphatase activity of Cn.
RANKL	↑	Inhibits NKA indirectly by increasing osteoclast number, bone resorption and Pi release
ALP	↑	ALP regulates Pi release, thereby indirectly inhibiting NKA activity. ALP activity in turn stimulated by Calcitonin.
TGF-β1	↑	Inhibits NKA activity. Calcitonin has been found to be a potent stimulator of TGF-β1 protein synthesis as well as TGF-β1 mRNA expression.
CT	↑	Inhibits NKA activity indirectly by upregulating TGF-β1 and ALP activity.
DA	↑	Inhibits NKA activity. PKC and cAMP signalling further contribute to dopaminergic inhibition of NKA.
TRH	↑	Inhibits NKA activity indirectly by inducing DA release.
PTH	↑	Inhibits NKA activity directly. PTH also inhibits NKA activity indirectly through activation of PKC, cAMP, PLA2 and PKA dependent pathways.
PLA2	↑	Inhibits NKA activity.
PGE2	↑	Inhibits NKA activity.
BgL	↑	Inhibits NKA activity, via activation of PKC, PLA2 and PGE2.
AGEs	↑	Inhibits NKA activity.
TSH	↑	TSH induces cAMP production and cAMP inhibits NKA activity by reducing ATP bioavailability and enhancing phosphorylation of the alpha-1 subunit of NKA

Abbreviations: NKA: Na^+^, K^+^-ATPase; PKC: Protein kinase C; ATP: Adenosine-triphosphate; cAMP: cyclic adenosine-monophosphate monophosphate; Cn: Calcineurin; CaM: Calmodulin; Mn2+: Magnesium; cGMP: Cyclic guanosine monophosphate; ENO1: Enolase; NO: Nitric oxide; Pi: Inorganic phosphate; ALP: Alkaline phosphatase; TGF-β1: Transforming growth factor β 1; CT: Calcitonin; DA: Dopamine; PTH: Parathyroid hormone; PLA2: Phospholipase A2; AA: Arachidonic Acid; Prostaglandin E2; BgL: Blood glucose; Glc: Glucose; RAGE: Receptors for advanced glycation end products; OC: Osteocalcin; DA: Dopamine; PTH: Parathyroid hormone; INS: Insulin;. TSH: Thyroid stimulating hormone; TRH: Thyroid-releasing hormone.

## Abbreviations

AA	Arachidonic Acid
ATP	Adenosine-triphosphate
ALP	Alkaline phosphatase
cAMP	cyclic adenosine-monophosphate monophosphate
CaM	Calmodulin
cGMP	Cyclic guanosine monophosphate
Cn	Calcineurin
COPD	Chronic obstructive pulmonary disease
CT	Calcitonin
F	Fluoride
Mn	Magnesium
NKA:	Na^+^, K^+^-ATPase
NO	Nitric oxide
PKC	Protein kinase C
Pi	Inorganic phosphate
PGE2:	Prostaglandin E2
PTH	Parathyroid hormone
PLA2	Phospholipase A2
RAGE	Receptors for advanced glycation end products
TGF-β1	Transforming growth factor β 1
TSH	Thyroid-stimulating-hormone, also called Thyrotropin
α	Alpha
β	Beta
U.S.A	United States of America
